# Bovine Milk Microbiota: Comparison among Three Different DNA Extraction Protocols To Identify a Better Approach for Bacterial Analysis

**DOI:** 10.1128/Spectrum.00374-21

**Published:** 2021-09-22

**Authors:** Paola Cremonesi, Marco Severgnini, Alicia Romanò, Lorenza Sala, Mario Luini, Bianca Castiglioni

**Affiliations:** a Institute of Agricultural Biology and Biotechnology, National Research Council, Lodi, Italy; b Institute of Biomedical Technologies, National Research Council, Segrate, Italy; c Istituto Zooprofilattico Sperimentale della Lombardia e Dell’Emilia-Romagna, Lodi, Italy; d Agroscope, Research Division, Food Microbial Systems, Bern, Switzerland; Broad Institute

**Keywords:** bacterial DNA extraction, milk microbiome, bulk tank milk, mock community

## Abstract

The bovine udder is colonized by a huge quantity of microorganisms that constitute the intramammary ecosystem, with a specific role in modulating not only udder homeostasis and mastitis susceptibility, but also the quality of the dairy products. However, generating high-quality bacterial DNA can be critical, especially starting from a complex biological matrix like milk, characterized by high fat, protein, and calcium contents. Here, bacterial DNA was recovered from a commercial ultra-high-temperature (UHT) milk sample artificially spiked with a predetermined mock community composition and from three bulk tank milk (raw milk) samples. The DNA was isolated using three different protocols to evaluate the effect of the extraction procedures on the milk microbiota composition. In the mock community experiment, the bacterial profiles generated by the three DNA extraction protocols were profoundly different, with the genera Staphylococcus, *Lactobacillus*, *Listeria*, and Salmonella underestimated by all the protocols. Only one protocol revealed values close to the expected abundances for Escherichia/*Shigella* spp., *Bacillus* spp., *Enterococcus* spp., and Pseudomonas spp. On the other hand, the nonspiked UHT milk sample exhibited a similar microbiota composition, revealing the prevalence of Acinetobacter spp., for all the DNA extraction protocols. For the raw milk samples, the three DNA extraction kits performed differently, revealing significant separations in both the microbial richness (alpha diversity) and composition (beta diversity). Our study highlights the presence of significant differences among these procedures, probably due to the different DNA extracting capacities and to the different properties of the milk samples, revealing that the selection of DNA extraction protocol is a critical point.

**IMPORTANCE** The advance of high-throughput technologies has increased our knowledge of the world of microorganisms, especially of microbial populations inhabiting living animals. This study provides evidence that milk, as other complex sources, could be critical for generating high-quality DNA for microbiota analysis. In addition, it demonstrates that the microbial population highlighted by metagenomic studies changes in relation to different DNA extraction procedures, revealing that attention should be paid especially when comparing different studies.

## INTRODUCTION

The advance of high-throughput technologies has increased our knowledge of the world of microorganisms, especially of microbial populations inhabiting living animals. For many years, microorganisms have been studied in terms of their morphological and growth characteristics. Thanks to the meta-omics sciences, the identification of dominant and subdominant microbes and their dynamics in overly complex ecosystems (1, 2), including milk, has been feasible. As recently described (3), the initial microbial load in raw milk from the healthy udder is low; then, in the passage from cow to bulk tank, there is an increase of microorganisms constituting the final milk microbiota. The potential sources can be due to complex microbial environments, such as the bedding material, the milking equipment, the milker’s hand, or cross suckling (4). These microorganisms play a specific role in modulating the udder homeostasis and mastitis susceptibility and also, together with those acquired during the milking procedure, in influencing the quality of the dairy products (4, 5). However, generating high-quality bacterial DNA could be critical, especially starting from a matrix with high fat, protein, and calcium constituents (6). These factors act as PCR inhibitors and can compromise the amplification of DNA. Methods for DNA isolation from raw milk and dairy products have been previously published to evaluate bacterial growth and distribution in raw whole milk, fat globules, milk fat, and casein pellets (6, 7). Normally, after casein precipitation, the isolation of bacterial DNA from milk samples is performed using academic protocols (7–9) or commercial kits including a bead-beating treatment (10, 11). The procedure usually consists of mechanical homogenization using microbeads at high speeds, treatments with buffers, detergents, or enzymes, and a lysis step (7). However, although the DNA isolation protocols from milk and dairy products generate good amounts of DNA suitable for PCR amplification starting from mastitic samples, they need to be improved for more efficient isolation of DNA from healthy milk, due to its typically low bacterial load. In the present study, three different protocols for bacterial DNA extraction (two commercial kits and a previously published protocol; protocols 1 to 3 [PR1 to PR3]) were compared in order to verify the influence of the extraction procedures on the milk microbiota composition. The two commercial kits are column-based systems, which use mechanical lyses and inhibitor removal technology. The published protocol (8), on the other hand, is based on the use of silica particles, with a chaotropic agent for cell lysis. The performance and reproducibility of these protocols were assessed based on the quality and quantity of the DNA extracted and the bacterial community composition by performing 16S rRNA-based high-throughput sequencing on an ultra-high-temperature-treated (UHT) milk sample spiked with a defined mock community and three raw milk samples.

## RESULTS

### DNA amount and purity.

The average DNA yield and purity were analyzed before performing the analysis of the milk microbiota, for both the UHT and raw milk samples. There was a significantly (*P* < 0.001, one-way analysis of variance [ANOVA] test) higher yield of DNA using PR1 compared to PR2 and PR3 in extracting DNA from both the raw and UHT milk; there was no statistically significant difference in the mean DNA yield between PR2 and PR3. On average, the PR1 DNA yield was 4.7- and 5.9-fold higher than that for PR2 and PR3, respectively, with raw milk and 2.8-fold higher than both PR2 and PR3 with the UHT milk samples (Table S1). The *A*_260/280_ ratio was higher (2.0) for PR2 with respect to those for PR1 and PR3 (1.5 for both).

### Microbiota analysis.

The microbiota structure of the UHT milk sample with the mock community added (21 sample units; 3 extraction protocols × 7 replicates each) was characterized by a total of 1,114,186 high-quality reads, with a mean of 53,056 ± 28,109 reads per sample. Similarly, the UHT milk sample without the mock community (21 sample units; 3 extraction protocols × 7 replicates each) had nearly the same yield (1,192,464 high-quality reads total; mean, 56,784 ± 33,081 reads). On the other hand, the microbiota profiling of the raw milk samples (45 sample units; 3 extraction protocols × 3 milk samples × 5 replicates each) displayed a higher yield (5,839,589 high-quality reads total; mean, 129,769 ± 64,021 reads). Operational taxonomic unit (OTU) rarefaction curves based on the Chao1 metric reached a plateau after about 20,000 reads (data not shown), suggesting that this depth of coverage was enough to describe the biological diversity within the samples. Thus, 3 sample units (one PR2 nonspiked sample unit and two PR3 spiked sample units) were discarded because their depth of coverage was below the threshold of 20,000 high-quality reads.

The microbial profiles were evaluated (i) along the mock and non-mock communities spiked into the UHT milk samples; (ii) along the three different DNA extraction protocols (PR1, PR2, and PR3); and (iii) along the three raw milk samples.

**FIG 1:**
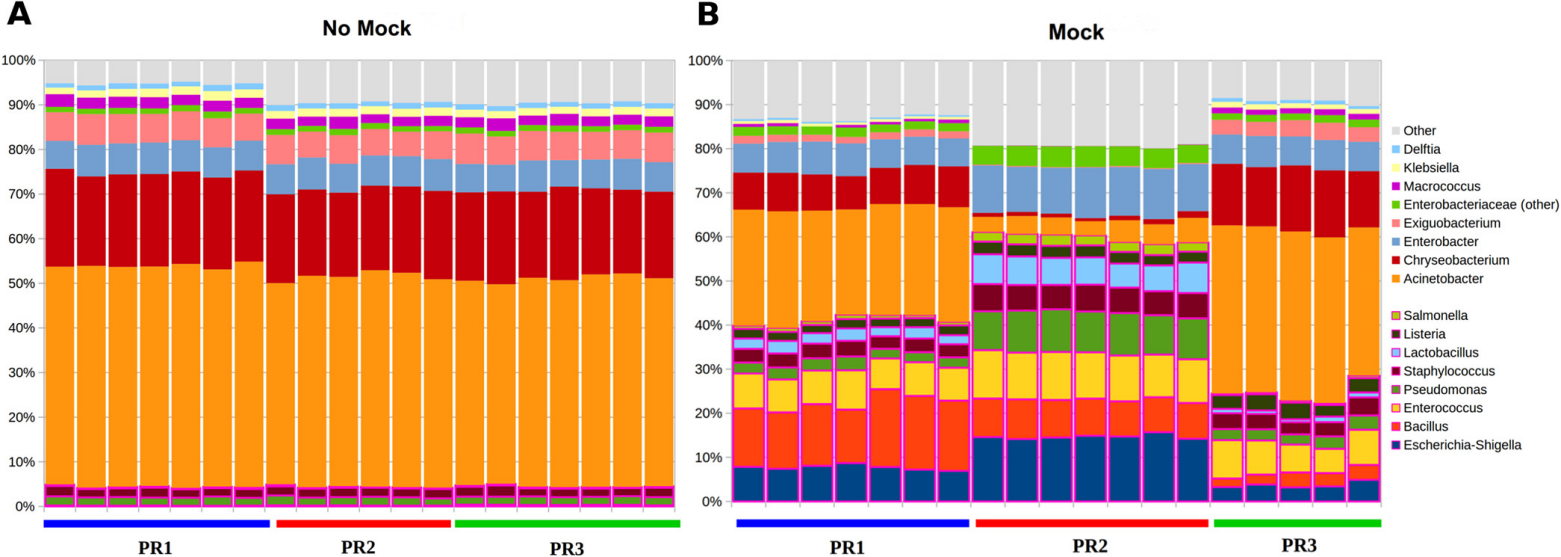
Microbiota profile for the spike-in experiment. The bar plots represent the relative abundance at the genus level for nonspiked (A) and spiked (B) UHT milk sample units. The colored bars under the plots group the replicates deriving from the same extraction procedure. For visualization purposes, members of the mock community are represented at the bottom of the bar plots in magenta-colored boxes.

The microbial profiles obtained were first evaluated with a comparison between the mock- and non-mock conditions in the UHT milk samples. The spike-in samples were constituted by adding to UHT milk with a known amount of a mock community, made up of bacterial species commonly not present or present in small amounts in milk. Independently of the DNA extraction protocols, the metagenomic analysis of the UHT milk sample without a mock community (*n* = 20; 7 PR1, 6 PR2, and 7 PR3) showed a similar microbiota composition (Fig. 1A). No significant variations were found between replicates.

For the nonspiked UHT milk sample, alpha-diversity (*P* = 1.0, permutation-based *t* test, for all diversity metrics; Fig. S1A in the supplemental material) and beta-diversity (*P* > 0.05 for all pairwise comparisons, unweighted UniFrac, Adonis test; Fig. S1B in the supplemental material) analyses did not show different microbial diversity among the three DNA extraction methods. The microbiota profile (Fig. S1C in the supplemental material) was dominated by Acinetobacter spp. (average relative abundances [avg rel ab], 49.7%, 49.5%, and 48.9% for PR1, PR2, and PR3, respectively), *Chryseobacterium* spp. (avg rel ab, 20.6%, 20.2%, and 20.7%, respectively), Enterobacter spp. (avg rel ab, 6.8%, 6.9%, and 6.8%, respectively), and *Exiguobacterium* (avg rel ab, 6.6%, 6.5%, and 6.7%, respectively); *Macrococcus*, Staphylococcus, Pseudomonas, and Klebsiella were subdominant genera whose average relative abundances were within ±0.1%.

After spiking with the mock community (*n* = 19; 7 PR1, 7 PR2, and 5 PR3), three diverse bacterial profiles were generated from the three different DNA extraction protocols (Fig. 1B). Using PR1, there was a predominance of Acinetobacter spp. (avg rel ab, 27.6%) but at a lower relative abundance with respect to UHT milk without the mock community, followed by *Bacillus* spp. (15.9%), *Enterococcus* spp. (8.4%), Escherichia/*Shigella* spp. (8.4%), and Enterobacter spp. (7.4%). A similar profile was observed for PR3 with the predominance of Acinetobacter (38.5%); a more equal distribution was observed among *Chryseobacterium* (14.6%), Enterobacter (7.0%), and *Enterococcus* (7.5%). Profoundly different results were obtained using PR2, with the lowest presence of Acinetobacter spp. (5.0%) and *Chryseobacterium* spp. (1.2%) with respect to the non-mock-community condition. On the other hand, PR2 estimated higher abundances of Escherichia/*Shigella* spp. (17.2%), Enterobacter spp. (12.7%), *Enterococcus* spp. (12.2%), and Pseudomonas spp. (10.9%).

**FIG 2:**
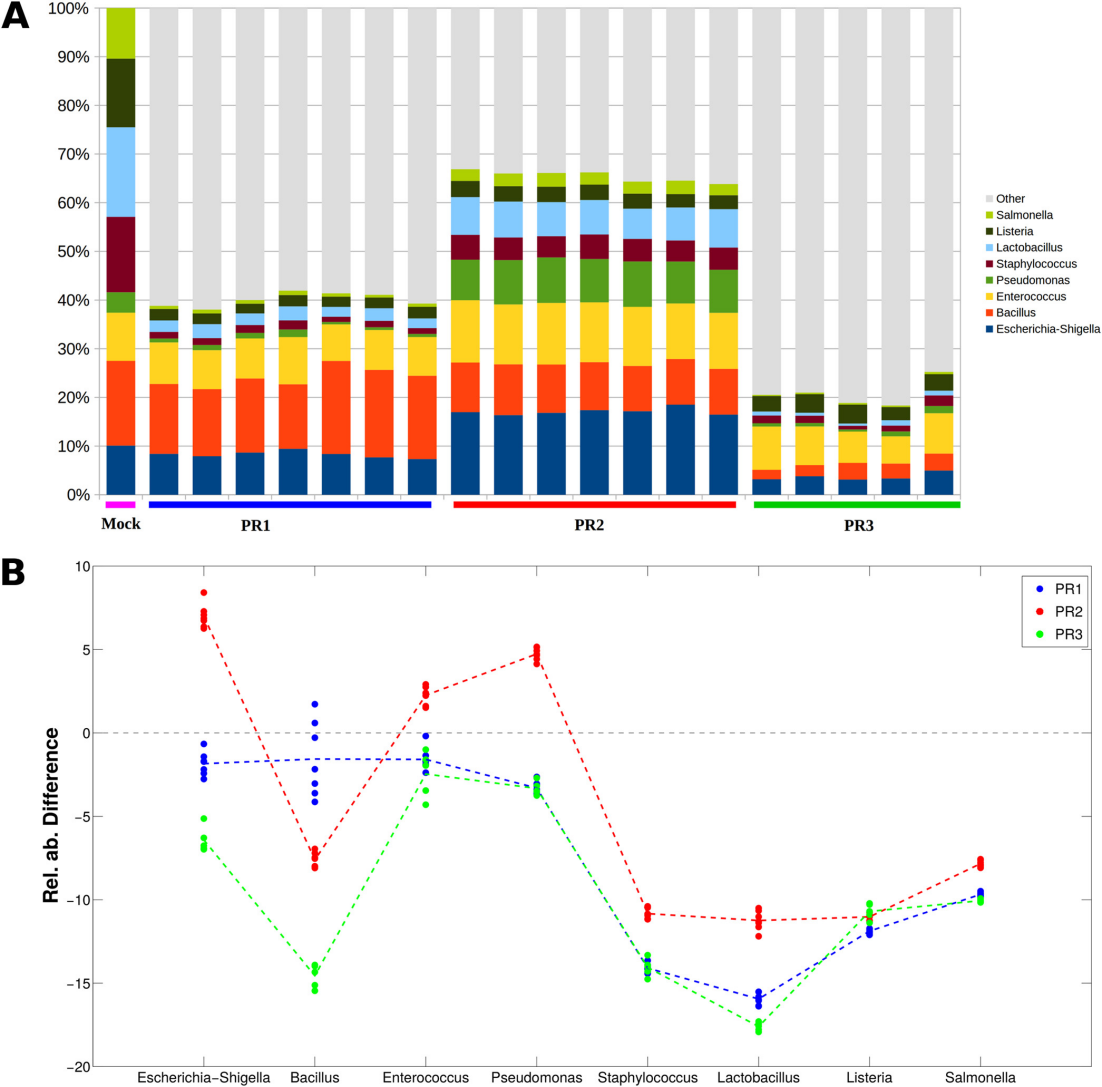
(A) Bar plot of the microbial average relative abundance for the mock-community-spiked sample units for the three extraction protocols. On the left, the theoretical composition, according to the producer’s specifications, is reported. (B) Line plot reporting the difference between the theoretical and actual composition of the milk samples for each of the three extraction protocols; the dots represent single replicated sample units, whereas the dashed line depicts the average difference.

In order to determine which was the “background,” due to the natural presence of the mock genera in the nonspiked UHT milk sample, the average of the relative abundance of the eight bacterial genera composing the mock community was calculated (Table S2). Before the mock-community spiking, the UHT milk samples revealed the presence of a certain amount of Staphylococcus spp. (mean values, 2.0%, 2.1%, and 2.1% for PR1, PR2, and PR3, respectively) and Pseudomonas spp. (mean values, 1.9%, 2.0%, and 1.9% for PR1, PR2, and PR3, respectively), while the remaining genera were irrelevant (values for all protocols, <0.2%). This was the optimal condition to say that what we were seeing in the spiked-milk sample was due to the mock community itself and that the contribution of the endogenous bacterial microbiota was negligible. The results obtained after a comparison between the theoretical composition of the mock community (eight bacterial genera distributed as reported in the first bar on the left of Fig. 2A) and the milk microbial composition with the three different extraction kits are reported in Fig. 2A; the background was subtracted from each sample. The results displayed that the three extraction procedures did not have the same performances (Fig. 2A). Among the eight genera of the mock community, four genera were underestimated by all the DNA extraction protocols (Fig. 2B). For the remaining four genera (Escherichia/*Shigella* spp., *Bacillus* spp., *Enterococcus* spp., and Pseudomonas spp.), PR3 consistently underestimated the content in the sample (with the relative abundance of *Bacillus* greatly underestimated, by −14.6% on average), while PR2 had the nearly opposite behavior, overestimating Escherichia/*Shigella* spp. (+7.0% on average), *Enterococcus* spp. (+2.3% on average), and Pseudomonas spp. (+4.7% on average). Finally, PR1 showed values closest to the expected value of the mock community for Escherichia/*Shigella* spp., *Bacillus* spp., and *Enterococcus* spp. (Fig. 2B) with relative abundance differences of −1.9%, −1.6%, and −1.6%, on average, respectively. Saccharomyces cerevisiae and Cryptococcus neoformans, both yeasts included in the mock community, could not be analyzed because they could not be amplified by the V3-V4 16S rRNA specific primers.

**FIG 3:**
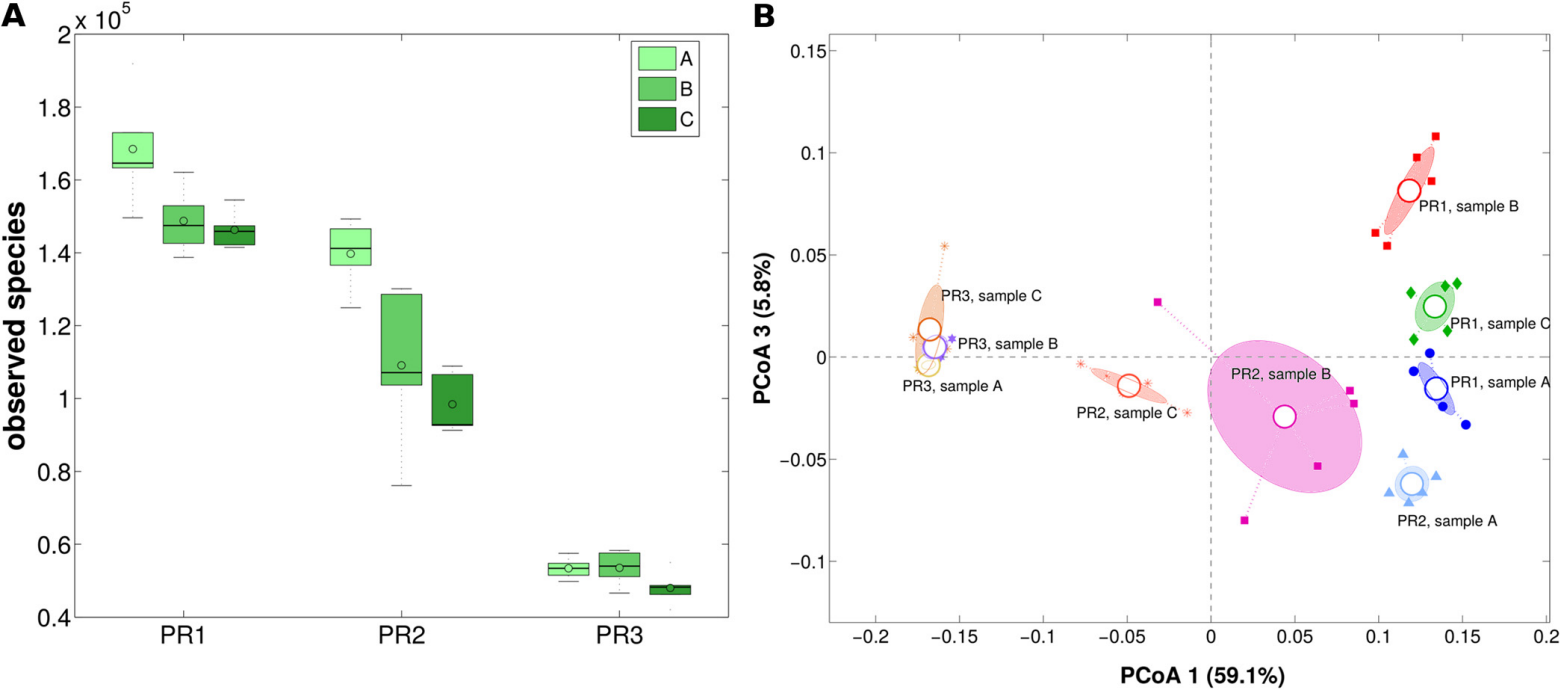
Raw milk microbiota profile. (A) Box plots of alpha diversity and (B) PCoA of unweighted Unifrac distances grouped by extraction protocol and milk sample. Each point represents a sample unit; the ellipses are the standard error of the mean (SEM)-based confidence intervals, and the colors indicate the extraction protocol-milk sample combinations. The first and third principal coordinates are represented.

The microbiota analysis of the raw milk samples collected from the three farms (A, B, and C) showed different behavior. Independently from the milk sample, alpha-diversity analysis (*P* = 0.003, permutation-based *t* test, observed species metrics for all comparisons), expressing the number of species in a specific ecosystem, revealed a different microbial diversity in relation to the DNA extraction procedure used, with the highest diversity for PR1 and the lowest for PR3 (Fig. 3A). On the other hand, the three raw milk samples extracted using the same protocol had similar biodiversity estimations (*P* > 0.05, permutation-based *t* test on all metrics). Beta-diversity analysis, on both weighted and unweighted UniFrac distances, helped further refine these results. A statistically significant difference (*P* = 0.001, Adonis test, weighted UniFrac distance for all pairwise comparisons) in the bacterial profiles among the DNA extraction procedures was revealed. The three raw milk samples clearly differed in composition of microbial taxa, as expected due to the different origins of the farms (*P* ≤ 0.01, Adonis test; Fig. 3B, Fig. S2 in the supplemental material).

**FIG 4:**
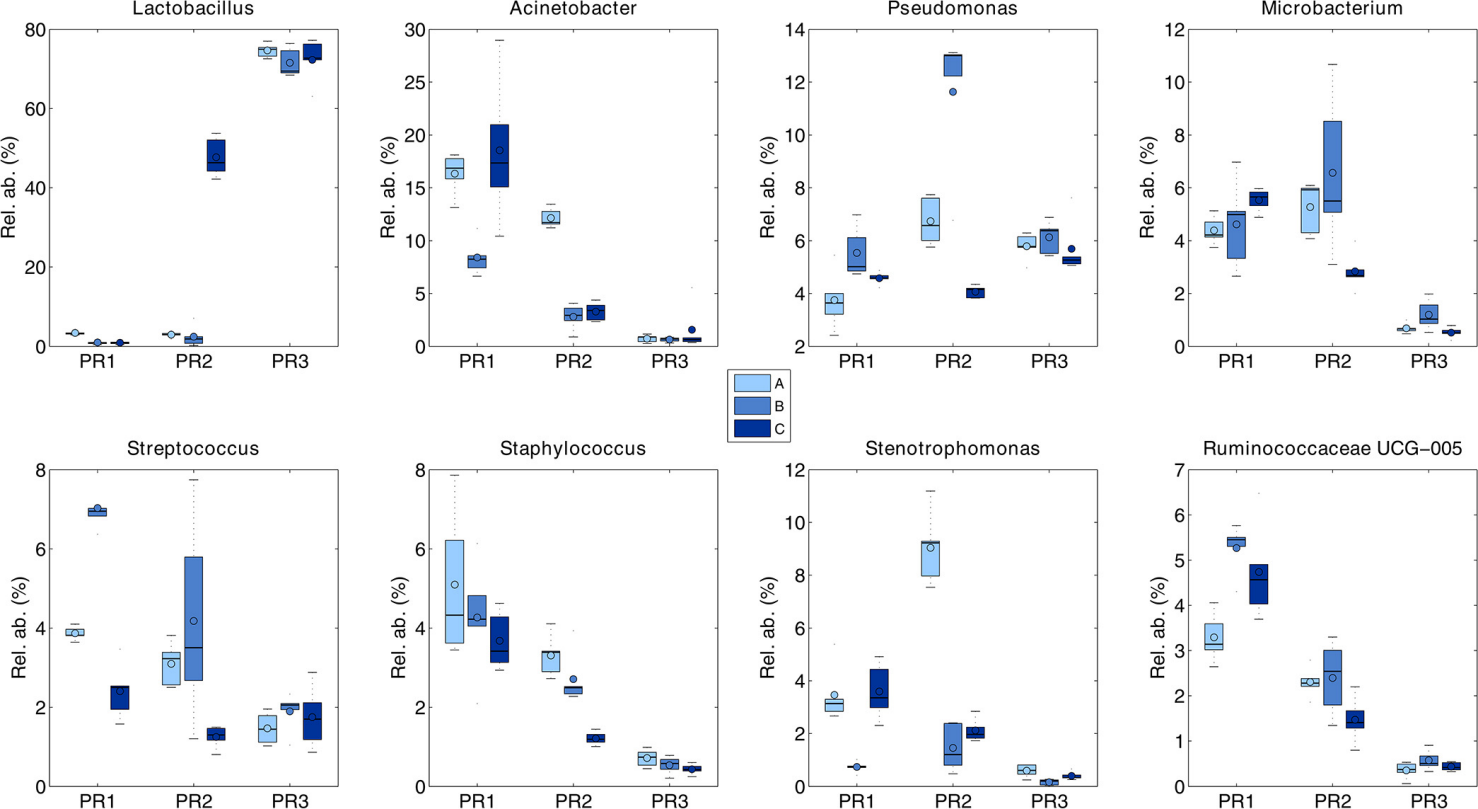
Box plots depicting the average abundance of the 8 main bacterial genera found in the raw milk samples, grouped by extraction protocol and milk sample.

A focus on the main eight genera (i.e., *Lactobacillus*, Acinetobacter, Pseudomonas, *Microbacterium*, Streptococcus, Staphylococcus, *Stenotrophomonas*, and *Ruminococcaceae* UCG-005 spp.), constituting more than 57% of the total bacterial relative abundance present in the raw milk samples collected from the three different farms, is reported in Fig. 4, confirming the extreme variability observed. For example, two DNA extraction protocols (i.e., PR1 and PR2) revealed a low relative abundance (<10%) of *Lactobacillus* spp., whereas PR3 had a consistently higher abundance (about 75%) for all the samples collected.

## DISCUSSION

As previously described (12), milk can be a routine source for bacterial genomic DNA in lactating dairy cattle, but the production of high-quality bacterial DNA could be a critical step when starting from such complex matrices. In this study, we compared three different DNA extraction methods (one published protocol [PR1] and two common commercial kits [PR2 and PR3]) for 16S rRNA-based microbiome profiling, assessing the impact of the DNA extraction methodology on the characterization of bovine milk microbiota. PR1 is a protocol previously used for the microbiota analysis of healthy milk samples (13, 14) and of dairy products, such as Trentingrana and Silter PDO cheeses (15, 16). To our knowledge, PR2 is predominantly utilized for different substrates, such as soil or hindgut microbiota characterization (17, 18), while PR3 is applied for bacterial profiles of bovine mastitic milk and/or healthy quarters (19) or for the characterization of bovine milk samples with low bacterial load (20).

Our findings revealed that the DNA yield was significantly influenced by the DNA extraction procedure. PR1 showed the highest DNA concentration, despite a lower *A*_260/280_ absorbance ratio, while PR3 had a low DNA extraction efficiency for raw bovine milk, which may in part be caused by the volume used for the analysis, the cell disruption during the first steps, and the binding capacity of DNA.

First, in this study, we evaluated the milk microbiome of a UHT milk sample spiked with a mock community with a defined composition and abundance of known microbes, namely, five Gram-positive and three Gram-negative bacteria. This approach was previously used for studying the DNA extraction procedures from different substrates, such as human milk (21), fecal samples (22), and low-biomass bovine milk samples (20). The first step of DNA extraction, involving bead beating or chemical lysis of the bacterial membranes, could influence the retrieval of specific bacterial taxa due to the procedure’s ability to disrupt the cell wall structure (23). Gram-positive microbes are usually more difficult to lyse (21). In our study, the 16S rRNA content of the spiked UHT milk sample was largely due to the mock community. Differently from a previous work (21), we obtained the closest proportional representation for *Bacillus* spp. (with PR1) and *Enterococcus* spp. (with all the tested protocols) to the mock community, despite an under-representation of similarly hard-to-disrupt bacteria like those of the genera Staphylococcus, Streptococcus, and *Listeria*. A similar result for Streptococcus spp. was obtained by Douglas and coworkers (24) in their evaluation of the influence of different extraction methods on the human milk microbiota profile. Moreover, PR3 introduced some biases in the distribution of taxa with an underestimation of all the mock-community bacteria, regardless of their Gram classification. This effect might be related to the DNA extraction method, which could influence the proportions of the taxa within the mock community, as described by Dahlberg and coauthors (20) during their analysis of a mock community containing Escherichia coli, Klebsiella pneumoniae, Streptococcus dysgalactiae, Staphylococcus aureus, and Trueperella pyogenes at three different dilutions. Moreover, PR1, with the lipid removal step prior to the DNA extraction procedure and the use of guanidine thiocyanate solution for the bacterial wall lysis, most closely recreated the mock-community representation for Escherichia coli, Bacillus subtilis, Enterococcus faecalis, and Pseudomonas aeruginosa. PR3, used by Quigley and coauthors, turned out to be successful at extracting Gram-positive and Gram-negative DNA from raw milk. On the other hand, when this protocol was applied to bulk tank milk samples with a low bacterial load, the lowest diversity was obtained, as in the present study (7).

As previously described (25, 26), high-temperature processing, especially UHT treatment and mechanical homogenization, can have different effects on milk, mainly bacteria and bacterial spore inactivation, protein denaturation, and reduction in fat content by breaking up fat particles. These milk structure alterations could be an explanation for the similar relative bacterial abundances obtained for the nonspiked UHT milk sample, extracted using the three different protocols, with the microbiota dominated by Acinetobacter spp.

This genus, among other 25 genera identified in milk samples (5), is commonly detected in different areas throughout a farm, including teat surfaces, milking parlors, hay, air, and dust, similarly to other psychrotrophs, such as Pseudomonas and *Aeromonas* spp. (5). Our results were in accordance with a previous study (27) in which the authors determined the microbiota of bulk tank milk. The data showed that high bacterial counts were dominated by a single cold-adapted species with high growth rates at low temperatures, such as Acinetobacter spp., *Chryseobacterium* spp., Streptococcus spp., and Pseudomonas spp. Differences in species richness were, finally, obtained when the three methodologies were used for analyzing raw milk samples. In line with previously published studies (6, 13), healthy bovine raw milk samples have a bacterial population dominated by *Firmicutes* and *Proteobacteria*, followed by the phyla *Bacteroidetes* and *Actinobacteria*, independently from the DNA extraction procedures. At the genus level, the core microbiome was dominated by *Lactobacillus*, Acinetobacter, Pseudomonas, *Microbacterium*, Streptococcus, Staphylococcus, *Stenotrophomonas,* and *Ruminococcus*, but significant variations were observed in the relative abundances of many taxa. PR1 and PR2 yielded similar results especially for the genera *Microbacterium* and *Lactobacillus*, while PR3 overestimated the genus *Lactobacillus*, with a loss of the remaining genera.

### Conclusions.

This study underlines the importance of the choice of a suitable protocol for DNA extraction, as the method can influence the 16S rRNA gene profiles generated from complex matrices such as milk samples. Although not many samples were considered in this study, the sample size was sufficient to allow the presence of significant differences among these procedures, probably due to the different DNA-extracting capacities and to the different properties of the milk samples under investigation. These aspects should be further investigated. Thus, we believe that when a comparison is necessary across different studies, particular attention should be paid to the extraction method chosen, as this could cause differences observed in the community structure. These findings confirm that the selection of a protocol that efficiently extracts nucleic acids from as many of the microorganisms present as possible is a critical point.

## MATERIALS AND METHODS

### Mock community.

To determine the effects of different DNA extraction procedures, 25 μl ZymoBIOMICS microbial community standard cells (number D6300; Zymo Research, EuroClone S.p.A., Milan, Italy) was added to 20 ml ultra-high-temperature (UHT) commercial milk, collected from a local market. This mock community contained three Gram-negative bacteria (Pseudomonas aeruginosa, Escherichia coli, and Salmonella enterica), five Gram-positive bacteria (Lactobacillus fermentum, Enterococcus faecalis, Staphylococcus aureus, Listeria monocytogenes, and Bacillus subtilis), and two yeasts (Saccharomyces cerevisiae and Cryptococcus neoformans), as described in Table S3 in the supplemental material. After the mock-community spiking, DNA was extracted from seven replicates of this artificially contaminated UHT milk and from seven replicates of the same nonspiked milk using the three DNA extraction procedures (as described below) for a total of 42 sample units (3 DNA extraction procedures × 2 experimental conditions [mock and non-mock spiking] × 7 replicates).

### Raw milk samples.

For this study, 500 ml of three raw bulk tank milk samples (raw milk) were collected in sterile collection tubes from three different farms (A, B, and C) and examined to ensure that they were free of contagious pathogens such as Staphylococcus aureus, Streptococcus agalactiae, and Mycoplasma bovis, according to the guidelines of the National Mastitis Council (28). Samples were delivered to the laboratory at 4°C and frozen at −20°C for metagenomic analysis. Microbial DNA was extracted using the three different DNA extraction kits (as described below) from five replicates for a total of 45 sample units (3 DNA extraction procedures × 3 samples [A, B, C] × 5 replicates).

### Bacterial DNA extraction procedures.

Bacterial DNA was extracted from the three raw milk samples and from the UHT milk samples with and without the mock community by using (i) the protocol of Cremonesi and coworkers (8), developed for pathogen detection but also for bacterial characterization in milk samples (13, 29) (protocol 1 [PR1]), and two commercial kits, (ii) the NucleoSpin soil kit (Macherey-Nagel, Dueren, Germany) (protocol 2 [PR2]) and (iii) the DNeasy PowerFood microbial kit (Qiagen, GmbH, Hilden, Germany) (protocol 3 [PR3]).

For PR1, a sample pretreatment was used: 5 ml of a milk sample was centrifuged at 500 × *g* for 5 min at 4°C, the supernatant was discarded, and the pellet was resuspended with 1 ml of saline solution (NaCl 0.9%) and centrifuged at 500 × *g* for 5 min at 4°C. The supernatant was discarded; bacterial DNA was extracted from the samples as described previously (8). Sample lysis was obtained using a combination of a chaotropic agent (guanidium thiocyanate) with silica particles. For PR2 and PR3, extraction started from 500 μl of a milk sample, following the manufacturers’ instructions. Bead tubes and homogenizers were used according to the manufacturers’ protocols.

The DNA quality and quantity were assessed using an ND-1000 spectrophotometer (NanoDrop Technologies, Wilmington, DE, USA). The isolated DNA was stored at −20°C until use.

### Library preparation.

Bacterial DNA was amplified using primers described in the literature (30) which target the V3-V4 hypervariable regions of the 16S rRNA gene. All PCR amplifications were performed in 25-μl volumes per sample. A total of 12.5 μl Phusion high-fidelity master mix 2× (Thermo Fisher Scientific, Waltham, MA, USA) and 0.2 μl of each primer (100 μM) was added to 2 μl genomic DNA (5 ng/μl). Blank controls (i.e., no DNA template added to the reaction) were also included. A first amplification step was performed in an Applied Biosystems 2700 thermal cycler (Thermo Fisher Scientific). The samples were denatured at 98°C for 30 s, followed by 25 cycles with a denaturing step at 98°C for 30 s, annealing at 56°C for 1 min, and extension at 72°C for 1 min, with a final extension at 72°C for 7 min. The amplicons were cleaned using Agencourt AMPure XP beads (Beckman, Coulter, Brea, CA, USA), and libraries were prepared following the 16S Metagenomic Sequencing Library Preparation Protocol (Illumina, San Diego, CA, USA). The libraries obtained were quantified by real-time PCR using KAPA library quantification kits (Kapa Biosystems, Inc., MA, USA), pooled in equimolar proportions and sequenced in one MiSeq (Illumina) run with 2 × 250-bp paired-end reads.

### Microbiota profiling.

The raw 16S rRNA sequences were processed through a pipeline, including fragment rebuilding by PANDAseq (31) and quality filtering aimed at removing low-quality reads (i.e., showing stretches of bases with a Q score of <3 for more than 25% of their length). For computational reasons, a subset of 50,000 reads for each sample was randomly extracted. Bioinformatic analyses were conducted using the QIIME pipeline release 1.8.0 (30), clustering filtered reads into operational taxonomic units (OTUs) at the 97% identity level. In order to sort out putative chimeras, singleton OTUs (i.e., supported by fewer than 2 reads across all samples) were removed. Taxonomic assignment was performed via the RDP classifier (32) against the SILVA database release 132 (https://www.arb-silva.de/fileadmin/silva_databases/qiime/Silva_132_release.zip), with a 0.5 identity threshold. The data set was downsampled to the least sequenced sample in order to have a comparable picture of the taxonomic composition.

The alpha diversity, which estimates the microbial species diversity on a single sample scale, was measured using the Chao1, Shannon’s diversity, observed species, and Faith’s phylogenetic diversity indexes; on the other hand, weighted and unweighted UniFrac distances and principal coordinates analysis (PCoA) were used to represent the microbial community structure for beta-diversity analysis, which measures the variation of microbial communities between samples (33).

In order to estimate the proportion of bacteria constituting the mock community naturally present in the milk samples, we calculated the average relative abundance of the corresponding genera in the non-mock samples for each extraction protocol. This “background” was subtracted from the samples with mock-community spike-in when evaluating the theoretical and real bacterial composition of the samples.

### Statistical analysis.

In order to compare the yields of DNA extracted using the three protocols, one-way ANOVA was performed, followed by the Tukey *post hoc* test for multiple comparisons. To compare the alpha-diversity metrics, a nonparametric, permutation-based *t* test was used, whereas statistically significant differences in the beta diversity were determined by employing the Adonis function in the R package vegan v. 2.5-6 (https://CRAN.R-project.org/package=vegan), which partitions the distance matrix among sources of variation, performing a permutation test by pseudo-*F* ratios. A *P* value of 0.05 was considered the threshold for statistical significance.

### Data availability.

All sequence data have been deposited in the Sequence Read Archive (SRA) of the NCBI under BioProject accession number PRJNA728536.
