# Supplementary material for: Bovine Milk Microbiota: Comparison among Three Different DNA Extraction Protocols To Identify a Better Approach for Bacterial Analysis
Source: Microbiol Spectr. 2021 Sep 22;9(2):e00374-21. doi: 10.1128/Spectrum.00374-21 (PMC8557886; doi:10.1128/Spectrum.00374-21)
Supplement: SUPPLEMENTAL FILE 1 — Supplemental material. Download SPECTRUM00374-21_Supp_1_seq9.pdf, PDF file, 0.3 MB [file spectrum00374-21_supp_1_seq9.pdf]

Supplementary Figure 1. Microbiota profiling of non-spiked UHT milk sample units. Bacterial profile was evaluated on (A) alpha-diversity (“observed species” metric, via a boxplot), (B) average relative abundance at genus level and (C) beta-diversity, represented via Principal Coordinate Analysis (PCoA) of unweighted Unifrac distances, where each point represents a sample unit, ellipses are the SEM-based confidence intervals and colors indicate the extraction protocol.

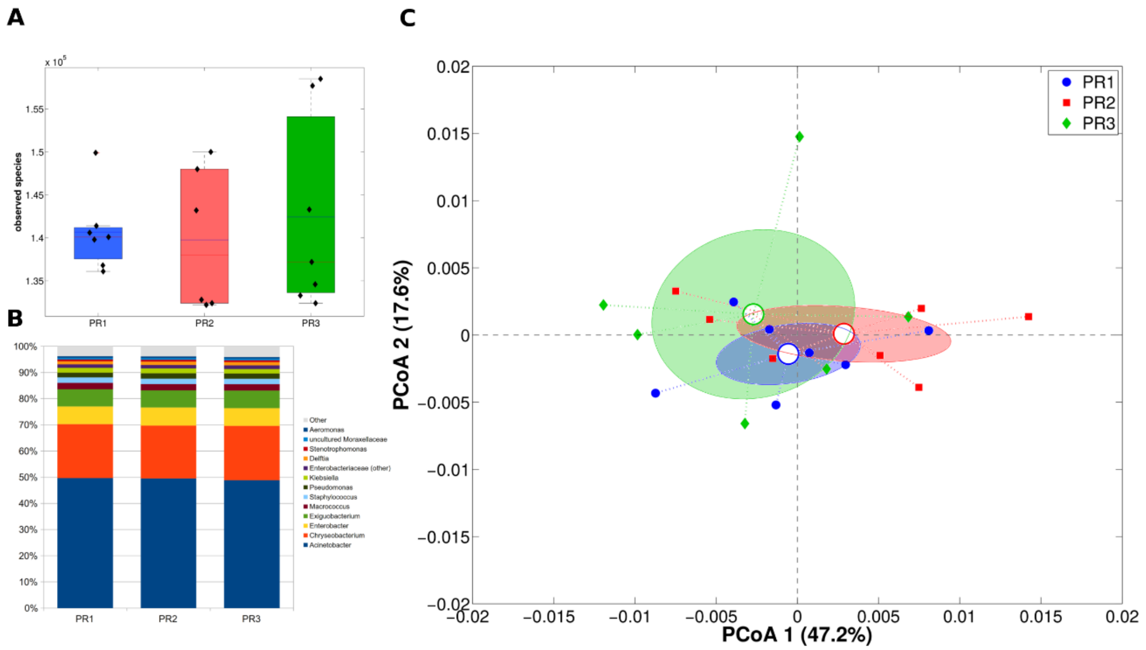

Supplementary Figure 2. PCoA of unweighted Unifrac distances grouped by milk sample. Each point represents a sample unit, ellipses are the SEM-based confidence intervals and colors indicate the milk sample. The second and third principal coordinates are represented.

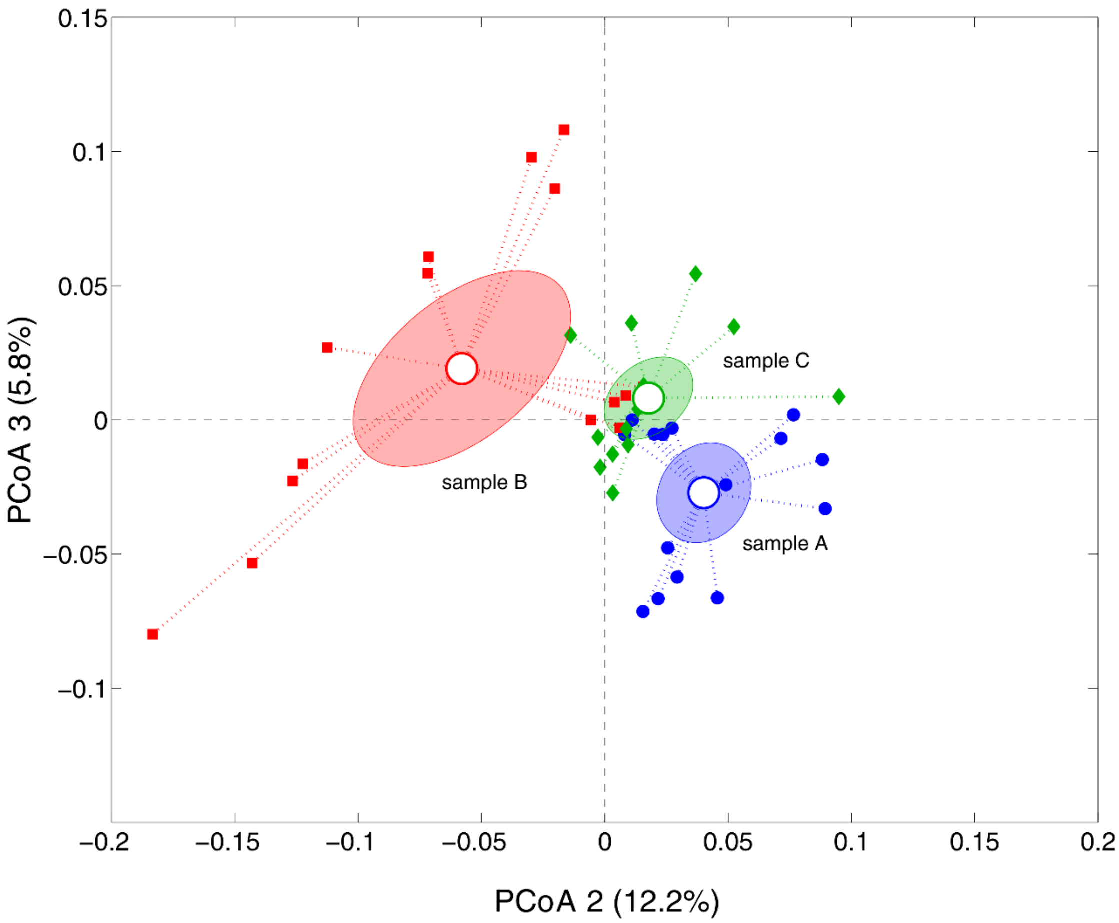

Supplementary Table 1. Table reporting the average DNA yield extracted from the raw and UHT milk samples analyzed in the study. For each protocol, we reported the mean yield and standard deviation. A one-way ANOVA was performed to compare the effect of three different extraction protocols on DNA yield.

			**Yield (ng/µl) Mean (st. dev.)**			**ANOVA p-values[2]**			
**Type**	**Milk sample**	**N[1]**	**PR1**	**PR2**	**PR3**	**Global**	**PR1-PR2**	**PR1-PR3**	**PR2-PR3**
Raw milk	A	5	23.8 (7.8)	5.3 (0.8)	3.3 (0.5)	<0.001*	<0.001*	<0.001*	0.770
	B	5	15.9 (6.9)	2.8 (3.6)	5.0 (2.3)	0.002*	0.002*	0.008*	0.741
	C	5	29.8 (7.2)	6.7 (0.8)	3.6 (1.4)	<0.001*	<0.001*	<0.001*	0.501
	All	15	23.2 (7.3)	4.9 (1.8)	3.9 (1.4)	<0.001*	<0.001*	<0.001*	0.810
UHT milk	mock	7	10.8 (3.0)	4.6 (0.1)	3.5 (0.8)	<0.001*	<0.001*	<0.001*	0.500
	no mock	7	8.5 (3.5)	2.7 (0.1)	3.5 (0.6)	<0.001*	<0.001*	0.001*	0.745

[1] number of replicates for each condition tested
[2] P-value of one-way ANOVA and Tukey's HSD Test for multiple comparisons. "*" indicates statistical significance ($p<0.05$).

Supplementary Table 2. Average relative abundance (standard deviation) of the 8 bacterial species composing the mock community in non-spiked UHT milk sample units. These data were used to estimate di “background” in the mock community analysis.

	Extraction protocol		
**Bacterial genera**	**PR1**	**PR2**	**PR3**
Escherichia-Shigella	0.11 (0.02)	0.13 (0.03)	0.19 (0.04)
Bacillus	0.02 (0.01)	0.03 (0.01)	0.06 (0.03)
Enterococcus	0.05 (0.02)	0.07 (0.02)	0.11 (0.01)
Pseudomonas	1.89 (0.14)	1.97 (0.21)	1.92 (0.08)
Staphylococcus	2.01 (0.18)	2.12 (0.05)	2.12 (0.28)
Lactobacillus	0.2 (0.03)	0.19 (0.07)	0.21 (0.04)
Listeria	0.01 (0.01)	0.01 (0.01)	0.02 (0.01)
Salmonella	0.17 (0.04)	0.17 (0.02)	0.15 (0.02)

Supplementary Table 3. Mock community composition as reported by Zymo Research (ZymoBIOMICS™ Microbial Community Standard Catalog No. D6300).

	Theoretical composition (%)				
**Species**	**Genomic DNA**	**16S only[1]**	**16S & 18S[1]**	**Genome copy[2]**	**Cell numbers[3]**
Pseudomonas aeruginosa	12	4.2	3.6	6.1	6.1
Escherichia coli	12	10.1	8.9	8.5	8.5
Salmonella enterica	12	10.4	9.1	8.7	8.8
Lactobacillus fermentum	12	18.4	16.1	21.6	21.9
Enterococcus faecalis	12	9.9	8.7	14.6	14.6
Staphylococcus aureus	12	15.5	13.6	15.2	15.3
Listeria monocytogenes	12	14.1	12.4	13.9	13.9
Bacillus subtilis	12	17.4	15.3	10.3	10.3
Saccharomyces cerevisiae	2	NA	9.3	0.57	0.29
Cryptococcus neoformans	2	NA	3.3	0.37	0.18

[1]The theoretical composition in terms of 16S (or 16S & 18S) rRNA gene abundance was calculated from theoretical genomic DNA composition with the following formula: 16S/18S copy number = total genomic DNA (g) × unit conversion constant (bp/g) / genome size (bp) × 16S/18S copy number per genome. Use this as reference when performing 16S targeted sequencing.

[2]The theoretical composition in terms of genome copy number was calculated from theoretical genomic DNA composition with the following formula: genome copy number = total genomic DNA (g) × unit conversion constant (bp/g) / genome size (bp). Use this as reference when inferring microbial abundance from shotgun sequencing data based on read depth/coverage.

[3]The theoretical composition in terms of cell number was calculated from theoretical genomic DNA composition with the following formula: cell number = total genomic DNA (g) × unit conversion constant (bp/g) / genome size (bp)/ploidy
